# miRNA Expression Profiling in G1 and G2 Pancreatic Neuroendocrine Tumors

**DOI:** 10.3390/cancers16142528

**Published:** 2024-07-13

**Authors:** Gábor Nyirő, Bálint Kende Szeredás, Ábel Decmann, Zoltan Herold, Bálint Vékony, Katalin Borka, Katalin Dezső, Attila Zalatnai, Ilona Kovalszky, Peter Igaz

**Affiliations:** 1 Department of Endocrinology, Faculty of Medicine, Semmelweis University, Korányi Str. 2/a, 1083 Budapest, Hungary; nyiro.gabor@semmelweis.hu (G.N.); szeredasbalint@gmail.com (B.K.S.); vekony.balint1997@gmail.com (B.V.); 2 Department of Internal Medicine and Oncology, Faculty of Medicine, Semmelweis University, Korányi Str. 2/a, 1083 Budapest, Hungary; 3 Department of Laboratory Medicine, Faculty of Medicine, Semmelweis University, Nagyvárad sq. 4., 1089 Budapest, Hungary; 4 Dr. László Vass Health Center, Municipality of District XV, 1152 Budapest, Hungary; decabel@gmail.com; 5 Division of Oncology, Department of Internal Medicine and Oncology, Semmelweis University, Baross Str. 23-25, 1082 Budapest, Hungary; 6 Department of Pathology, Forensic and Insurance Medicine, Faculty of Medicine, Semmelweis University, Üllői Str. 93, 1083 Budapest, Hungary; borka.katalin@semmelweis.hu; 7 Department of Pathology and Experimental Cancer Research, Faculty of Medicine, Semmelweis University, Üllői Str. 26, 1085 Budapest, Hungary; dezso.katalin@semmelweis.hu (K.D.); zalatnai.attila@semmelweis.hu (A.Z.); kovalszky.ilona@med.semmelweis-univ.hu (I.K.)

**Keywords:** pancreatic neuroendocrine tumor, grade, microRNA, biomarker, machine learning, formalin-fixed paraffin-embedded

## Abstract

**Simple Summary:**

Pancreatic neuroendocrine tumors are rare, but their incidence is rising. Several grades exist, and distinguishing between these is pivotal for clinical management. Currently, the grades can only be differentiated by histological analysis requiring invasive sampling. MicroRNAs are short non-protein coding RNA molecules that were shown to be differentially expressed in a wide variety of tumors. Here, we examined whether microRNAs could be exploited to differentiate grade 1 and 2 pancreatic neuroendocrine tumors and established significantly differentially expressed microRNAs.

**Abstract:**

Pancreatic neuroendocrine neoplasms pose a growing clinical challenge due to their rising incidence and variable prognosis. The current study aims to investigate microRNAs (miRNA; miR) as potential biomarkers for distinguishing between grade 1 (G1) and grade 2 (G2) pancreatic neuroendocrine tumors (PanNET). A total of 33 formalin-fixed, paraffin-embedded samples were analyzed, comprising 17 G1 and 16 G2 tumors. Initially, literature-based miRNAs were validated via real-time quantitative reverse transcription polymerase chain reaction (RT-qPCR), confirming significant downregulation of *miR-130b-3p* and *miR-106b* in G2 samples. Through next-generation sequencing, we have identified and selected the top six miRNAs showing the highest difference between G1 and G2 tumors, which were further validated. RT-qPCR validation confirmed the downregulation of *miR-30d-5p* in G2 tumors. miRNA combinations were created to distinguish between the two PanNET grades. The highest diagnostic performance in distinguishing between G1 and G2 PanNETs by a machine learning algorithm was achieved when using the combination *miR-106b + miR-130b-3p + miR-127-3p + miR-129-5p + miR-30d-5p*. The ROC analysis resulted in a sensitivity of 83.33% and a specificity of 87.5%. The findings underscore the potential use of miRNAs as biomarkers for stratifying PanNET grades, though further research is warranted to enhance diagnostic accuracy and clinical utility.

## 1. Introduction

Pancreatic neuroendocrine neoplasms (PanNENs) are among the most common neuroendocrine tumors and have shown an increasing incidence over the previous decades [1,2]. According to the most recent World Health Organization (WHO, 2022) classification system, pancreatic neuroendocrine tumors (PanNETs) and pancreatic neuroendocrine carcinomas (PanNECs) belong to the group of PanNENs. Well-differentiated PanNETs are grouped into grades 1 to 3 based on the Ki-67 index (<3%, 3–20%, and >20%, respectively), whereas poorly differentiated tumors are categorized as PanNECs [3]. PanNETs comprise functioning or hormonally active tumors, among others such as insulinoma, gastrinoma, glucagonoma, somatostatinoma, vasoactive intestinal peptide-secreting tumors (VIPomas), ACTH-producing tumors, and non-functioning, hormonally inactive tumors [4]. Hormone-producing tumors are associated with typical syndromes, whereas hormonally inactive tumors are often discovered incidentally.

Histologically, the differentiation between low/intermediate grade PanNETs (G1 and G2) and PanNECs is not specifically challenging, while distinguishing high-grade PanNETs (G3) from PanNECs can be rather difficult [5]. The differentiation of grade 1 from grade 2 PanNETs is also relevant. G1 tumors have a more favorable prognosis, with an approximately double median overall survival compared to G2 tumors [6]. Treatment strategies for G1 and G2 PanNETs are similar, but G2 tumors often require more aggressive treatment. However, the choice of treatment is influenced by the disease extent rather than by grade [7,8]. The primary treatment for non-metastatic tumors is usually surgical. Treatment options for differentiated metastatic PanNETs include somatostatin analogues, the mTOR inhibitor everolimus, the multikinase inhibitor sunitinib, peptide receptor radionuclide treatment, systemic chemotherapy, etc. [7,8,9]. Gallstone formation represents a potential long-term side effect of somatostatin analogue treatment [10] that could warrant cholecystectomy [11]. Moreover, the grade can change during the progression of the disease [12]. G1 and G2 PanNETs are distinguished histologically, requiring invasive sampling. There is no other reliable, accurate, minimally or non-invasive biomarker that could help in the differentiation, but such a biomarker would be clinically relevant.

Mature microRNAs (miRNA, miR) are small, non-coding, single-stranded RNA molecules, typically ranging from 19 to 25 nucleotides in length. They play a key role in regulating gene expression, primarily operating at the posttranscriptional level. MicroRNAs have tissue-specific expression and are secreted in body fluids as well [13]. Numerous studies have demonstrated the utility of miRNAs as valuable biomarkers across various diseases, encompassing a range of neoplastic conditions. As miRNAs can be found in the blood as well, they might be used as minimally invasive biomarkers. Recently, *hsa-miR-21*, *hsa-miR-10a*, and *hsa-miR-106b* were found to be upregulated in more proliferative PanNENs (G2 and G3) compared to grade 1 tumors by studying formalin-fixed, paraffin-embedded (FFPE) samples [14]. *miR-96-5p* and *miR-130b-3p* showed significantly lower expression in G1 compared to G2 to G3 gastroenteropancreatic NETs (GEP-NETs), and *miR-194-5p* showed a significant decrease through grades of GEP-NETs [15]. *miR-30d-5p*, *miR-451a*, and *let-7i-5p* showed decreasing trends of expression from G1 to G2 and G3 samples in a small cohort [16]. Significantly higher expression of circulating miRNA levels was also identified in PanNEN samples compared to healthy control samples (serum *miR-193b*) or chronic pancreatitis (*miR-21*) [17,18]. Significantly lower levels of serum *miR-1290*, *miR-584*, *miR-1285*, *miR-1825*, and *miR-550-002410* were found in PanNET samples compared to pancreatic adenocarcinomas [19]. To the best of our knowledge, there have only been three articles describing miRNAs that can differentiate between G1 and G2 (or G3) PanNETs so far [14,15,16]. In other tumors, miRNA combinations have been found to be superior to individual miRNAs in differentiating between different tumor types [20].

Our aim was to investigate the expression of tissue miRNAs in G1 and G2 PanNETs. We have studied certain miRNAs described in the literature previously, but also performed miRNA profiling to identify novel miRNAs.

## 2. Materials and Methods

### 2.1. Tissue Collection

A total of 33 histologically confirmed formalin-fixed, paraffin-embedded (FFPE) samples were utilized, all sourced from human tumor blocks (Table 1). Specifically, 17 grade 1 (G1) and 16 grade 2 (G2) PanNET samples were procured from the Pathology Departments of Semmelweis University, Budapest, Hungary. To ascertain tumor grade and identify the Region of Interest within the block, which contained “pure” tumor tissue of the specified grade, hematoxylin–eosin slides were prepared, marked, and assessed by expert pathologists. For RNA isolation, 4 × 20 µm micro-dissected sections were prepared and placed into RNase-free tubes. All procedures were conducted in compliance with the applicable guidelines and regulations (Ethical permission by the Hungarian Health Council IV-2388/1/2022/EKU).

### 2.2. Sample Processing and RNA Isolation

For the extraction of total RNA, encompassing miRNAs, the RecoverAll™ Total Nucleic Acid Isolation Kit for FFPE (Thermo Fisher Scientific, Waltham, MA, USA) was employed. Following deparaffinization using xylene isomers, the samples underwent digestion and purification as per the manufacturer’s instructions. As a spike-in control for purification efficiency and for external control in the real-time quantitative reverse transcription polymerase chain reaction (RT-qPCR) process, 2 μL of 5 nM *Syn-cel-miR-39* was added. The obtained total RNA was stored at −80 °C until further processing. RNA concentration was determined using the Qubit 4 Fluorimeter with the Qubit RNA Broad Range RNA Assay Kit (Thermo Fisher Scientific).

### 2.3. Real-Time Quantitative Reverse Transcription Polymerase Chain Reaction (RT-qPCR) for Quantification and NGS Validation

Initially, each miRNA was reverse-transcribed into cDNA individually using miRNA-specific primers (TaqMan miRNA assays; Thermo Fisher Scientific) in a ProFlex™ PCR machine (Thermo Fisher Scientific). Each RT sample included 10 ng of RNA as starting material. Subsequently, 1 µL of the synthesized cDNA was subjected to qPCR using miRNA-specific fluorescently labeled hydrolysis probes (TaqMan probes, Thermo Fisher Scientific). The Quantstudio 7 Flex Real-Time PCR System (Thermo Fisher Scientific) was used with a 96-well fast block, and Ct values were determined for each target. Relative quantification of miRNAs was performed utilizing *RNU 48* (ID: 001006) as an internal control target and *cel-miR-39* (ID: 000200) as an external control target, employing the ΔΔCt method as described by Livak and Schmittgen [21]. The internal and external control was unified into one measure by calculating the geometric mean of the two values. Specific primers for miRNAs selected based on literature research were [*miR-130b-3p* (ID: 000456); *miR-194-5p* (ID: 000493); *miR-106b* (ID: 000442); and *miR-96-5p* (ID: 000186)] and those identified via NGS were [*miR-127-3p* (ID: 000452); *miR-129-5p* (ID: 000590); *miR-769-5p* (ID: 001998), *miR-671-5p* (ID: 197646_mat); *miR-375-3p* (ID: 000564); and *miR-30d-5p* (ID: 000420)].

### 2.4. miRNA Expression Profiling from FFPE Samples by Next-Generation Sequencing (NGS)

For library preparation, 100 ng of total RNA served as the initial material, using the QIAseq miRNA Seq Kit (Qiagen GmbH, Hilden, Germany). Sequentially, adapters were ligated to both the 3′ and 5′ ends of miRNAs, followed by reverse transcription to create cDNA. The cDNA product was purified using magnetic bead separation. In the subsequent library amplification stage, cDNA was labeled with dual molecular indexes, each containing a unique sequence for individual samples. Following library amplification, further purification by magnetic beads was conducted. Each sample was then diluted to a concentration of 4 nM and equimolarly combined into a pooled cDNA library at a concentration of 4 nM. After chemically denaturing 5 µL of the pooled library and diluting it to 10 pM, sequencing was performed using the MiSeq v3 150 cycles sequencing kit on a MiSeq^™^ NGS sequencer (Illumina, San Diego, CA, USA). The acquired sequence data underwent demultiplexing and fastq files were analyzed using the Qiagen GeneGlobe system (QIAGEN RNA-seq Analysis Portal 5.0, (website: rnaportal.qiagen.com)). This software platform facilitates miRNA annotation, count determination, and gene expression analysis.

### 2.5. Statistical– and Machine Learning Analysis

GraphPad Prism version 6.01 (GraphPad Software, La Jolla, CA, USA) and R for Windows version 4.4.0 environment (R Core Team, 2024, R Foundation for Statistical Computing, Vienna, Austria) were used in the analysis of RT-qPCR data. As most of the RT-qPCR data were significantly skewed, outlier removal was performed prior to any statistical analysis. All the data that were over the upper quartile + 1.5 × interquartile range or were under the lower quartile − 1.5 × interquartile range were marked as NA. For differentiating between G1 and G2 PanNET groups, Welch’s *t*-test was used. *p*-Values of < 0.05 were considered significant. A 90–10% learner–tester cross-validation simulation with 100,000 iterations was run to differentiate G1 and G2 tumors utilizing a single hidden-layer neural network model (R package nnet, version 7.3-19). Each miRNA’s differentiation potential was tested as a standalone marker, as well as the potential of their combinations. The combination models included up to 6 miRNAs out of the 10 available, resulting in a total of 847 possible combinations to investigate. Following the simulation procedure, the original and predicted groups were compared, and the number of true negatives (TN), true positives (TP), false negatives (FN), and false positives (FP) were determined. As the last step, sensitivities (Se) and specificities (Sp) of the neural network models were calculated using the Se = TP/(TP + FN) and the Sp = TN/(TN + FP) equations, respectively. The best-performing model was selected for receiver operating characteristic (ROC) curve modeling, which was performed using the R package pROC (version 1.18.5).

## 3. Results

### 3.1. Real-Time Quantitative Reverse Transcription Polymerase Chain Reaction (RT-qPCR) Validation of miRNAs Found in Literature

**Figure 1:**
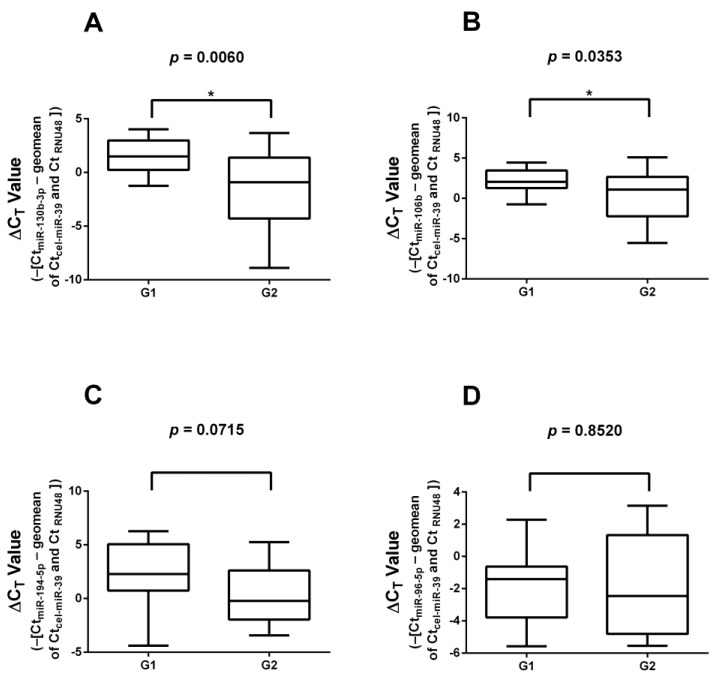
Expression of miRNAs identified by literature search. (**A**) *miR-130-3p;* (**B**) *miR-106b;* (**C**) *miR-194-5p;* (**D**) *miR-96-5p*. *miR-130b-3p* and *miR-106b* showed significantly lower expression in G2 samples compared to G1 samples. *: significant difference (*p* < 0.05).

Thirty-three FFPE samples (17 G1 and 16 G2) were subjected to RT-qPCR validation. The results of the validation of significantly differently expressed miRNAs found in the literature are presented in Figure 1. *miR-130b-3p* and *miR-106b* showed significantly lower expression in G2 samples compared to G1 tumors, while *miR-194-5p* and *miR-96-5p* showed no significant differences in expression between the two groups. No changes in the results were found when the insulinoma cases were removed.

### 3.2. miRNA Expression Profiling by Next-Generation Sequencing

Altogether, five G1 and five G2 FFPE samples were subjected to NGS profiling. miRNAs are listed in Table S1. From the sequenced miRNAs, we selected the six miRNAs showing the greatest difference between G1 and G2 samples (*miR-127-3p*, *miR-129-5p*, *miR-769-5p*, *miR-671-5p*, *miR-375-3p*, and *miR-30d-5p*). NGS data were uploaded to the openly accessible repository under the Gene Expression Omnibus accession identification number GSE265752.

### 3.3. RT-qPCR Validation of Significantly Differentially Expressed miRNAs

**Figure 2:**
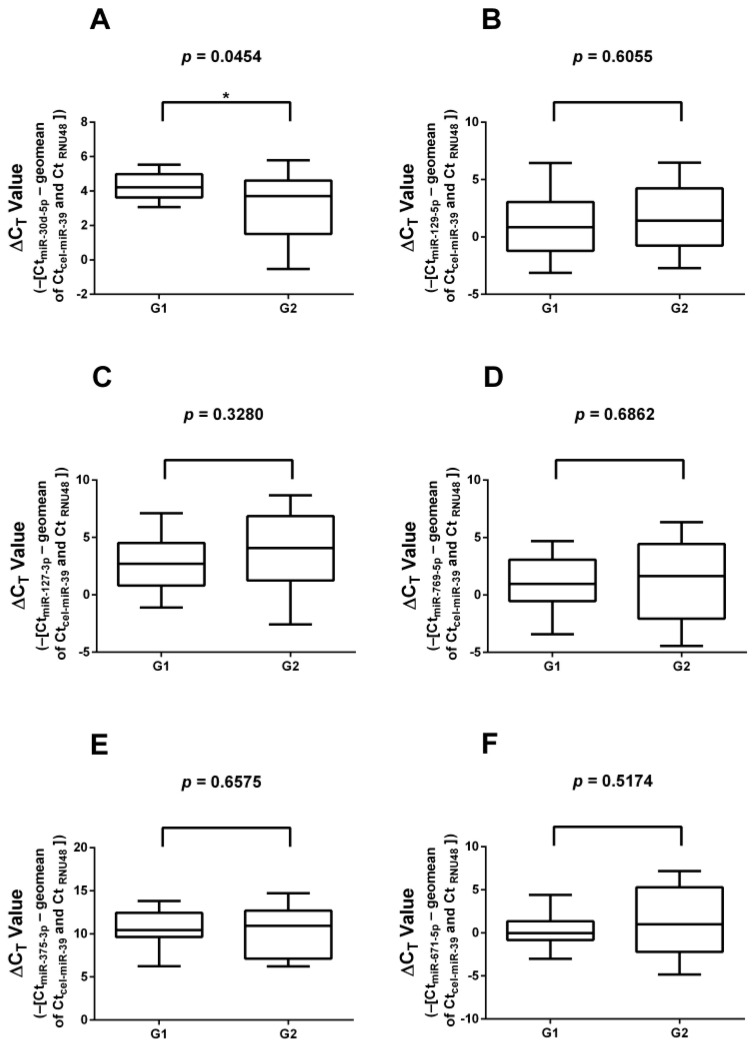
RT-qPCR validation of significantly differentially expressed miRNAs selected based on NGS data. (**A**) *miR-30d-5p*; (**B**) *miR-129-5p*; (**C**) *miR-127-3p*; (**D**) *miR-769-5p*; (**E**) *miR-375-3p*; (**F**) *miR-671-5p*. *miR-30d-5p* showed significantly lower expression in G2 samples compared to G1 samples. *: significant difference (*p* < 0.05).

The 6 differentially expressed miRNAs identified during NGS profiling were validated on all 33 G1 and G2 FFPE samples used for the validation of literature-based miRNAs (17 G1 and 16 G2 samples; Figure 2). Validation showed a significant downregulation of *miR-30d-5p* (*p* = 0.0454) in G2 samples relative to G1 samples. The other five selected miRNAs [*miR-127-3p* (*p* = 0.3280); *miR-129-5p* (*p* = 0.6055); *miR-769-5p* (*p* = 0.6862); *miR-671-5p* (*p* = 0.5174), and *miR-375-3p* (*p* = 0.6575)] displayed no difference in expression. When comparing insulinoma samples with non-insulinoma PanNET samples, no statistically different expression levels were found. Furthermore, no change in the results was found if all the insulinoma cases were removed.

### 3.4. Diagnostic Performance of miRNAs

**Figure 3:**
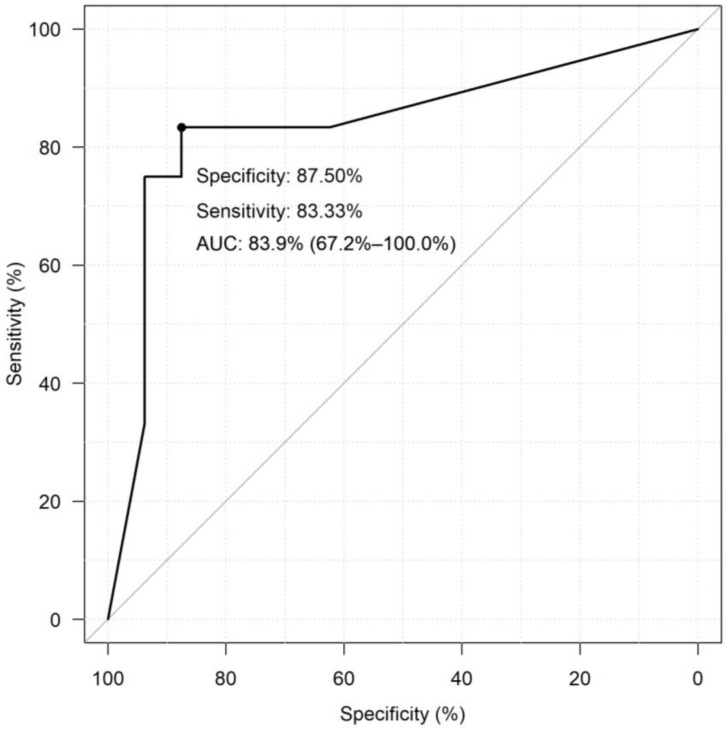
The receiver operator characteristic (ROC) curve of the best-performing miRNA combination. In the ROC model, the predictor was the percentage value estimated by the selected neural network model. The following simulation procedure was used to estimate the percentages for each sample. The best-performing neural network model was re-run by training a new learning sample omitting the sample selected for testing. We then performed model prediction on whether the given sample was Grade I or II. All samples were tested using 10,000 iterations. The sensitivity and specificity values differ from that of the data in Table 2 as this is a recalculation by ROC.

The diagnostic performance of selected and validated miRNAs was assessed using neural network models for all of the 847 miRNA combinations. The best-performing miRNA combination in differentiating G1 PanNETs from G2 PanNETs was *miR-106b + miR-130b-3p + miR-127-3p + miR-129-5p + miR-30d-5p* with a specificity of 77.44% and a sensitivity of 87.82% (Table 2). Only the ten best-performing combinations are shown here, while the complete dataset is included in Table S2. The diagnostic performance of the combination was better than that of individual miRNAs. The ROC curve of the best-performing miRNA combination resulting from the neural network model analyses is shown in Figure 3, where a sensitivity of 83.33 and specificity of 87.5% was achieved.

## 4. Discussion

We studied miRNAs showing differential expression between G1 and G2 PanNETs. First, we examined miRNAs already described to be differentially expressed between G1 and G2 GEP-NETs in the literature. From the selected four miRNAs, we were unable to demonstrate significant differences in the expression levels between the two tumor grade groups for *miR-96-5p* and *miR-194-5p*. *miR-106b* was confirmed to be decreased in G2 relative to G1 samples. However, the expression of *miR-130b-3p* showed an inverse change as reported previously, being underexpressed in G2 [15]. This incongruence might arise from the imparity of the original samples, namely that Cavalcanti et al. [15] used GEP-NETs, while we used only its subset, PanNETs. There are differences between pancreatic and other GEP-NETs, such as PanNENs being more likely to have a hereditary background (e.g., von Hippel–Lindau syndrome, multiple endocrine neoplasia syndrome type 1, tuberous sclerosis, and neurofibromatosis) or have distinct biological activity [3,22]. Moreover, the treatment protocols between PanNEN and small bowel NEN are also different [8,23]. 

We have discovered further significantly differentially expressed miRNAs between G1 and G2 PanNETs by NGS. From these miRNAs, we selected the top six miRNAs showing the largest differences. RT-qPCR validation of the selected miRNAs confirmed the significant downregulation of *miR-30d-5p* in G2 samples while for *miR-127-3p*, *miR-129-5p*, *miR-769-5p*, and *miR-671-5p*, no significant difference was detected. In the literature, *miR-127-3p* mainly functions as a tumor suppressor [24,25]; however, in our study, only non-significant changes in its expression in higher-graded PanNETs could be observed. It is not unusual for a miRNA to have tumor suppressor or oncogenic activity depending on the tissue [26]. *miR-127-3p* and *miR-375* appear to be involved in insulin secretion, and they are highly abundant in pancreatic islet cells [27]. The expression of *miR-127-3p* was non-significantly higher in our few insulinoma samples compared to non-functional PanNETs. *miR-127* was also found significantly overexpressed in PanNENs and their corresponding metastases compared to other GEP-NETs [28]. *miR-671* has been shown to be underexpressed in pancreatic ductal carcinoma among other tumors and upregulated in colorectal, prostate, and hepatocellular cancer [29]. miRNA combinations showed higher diagnostic performance than individual miRNAs in concordance with the literature [20].

## 5. Conclusions

In conclusion, we have found a miRNA combination, namely *miR-106b + miR-130b-3p + miR-127-3p + miR-129-5p + miR-30d-5p*, that can possibly differentiate between G1 and G2 PanNETs. The diagnostic performance of these miRNAs is not perfect but is promising. Further, preferably multicentric studies on larger sample cohorts are warranted to validate and increase the diagnostic accuracy of the combined miRNA marker. If matching circulating miRNAs could be identified, this might open the way for minimally invasive blood-borne biomarkers and would have major clinical relevance in determining the grade of PanNETs.

## Figures and Tables

**Table 1:** Characteristics of tumor samples studied. NF: non-functioning. M: male, F: female.

Sample No.	Tumor Grade	Ki-67 Index	Sex	Age	Hormonal Activity
1.	1	2%	M	60	NF
2.	1	2%	F	34	NF
3.	1	<1%	F	46	Insulinoma
4.	1	1%	F	42	NF
5.	1	1%	F	49	Insulinoma
6.	1	3%	F	70	NF
7.	1	0%	F	65	NF
8.	1	1%	M	69	NF
9.	1	<1%	F	67	NF
10.	1	2%	F	74	NF
11.	1	<1%	F	40	NF
12.	1	<1%	F	53	NF
13.	1	1%	F	44	Insulinoma
14.	1	<2%	M	70	NF
15.	1	<1%	M	71	NF
16.	1	<1%	F	79	NF
17.	1	1%	F	56	NF
18.	2	20%	F	48	NF
19.	2	15%	F	42	NF
20.	2	10%	M	69	NF
21.	2	5%	F	60	NF
22.	2	1–3%	F	62	NF
23.	2	10%	F	65	NF
24.	2	5%	M	66	NF
25.	2	1–4%	M	49	NF
26.	2	5–8%	M	44	NF
27.	2	2.8–3.1%	F	76	NF
28.	2	5–10%	M	54	NF
29.	2	5%	M	47	NF
30.	2	5%	M	48	NF
31.	2	20%	F	49	NF
32.	2	5%	F	74	NF
33.	2	7%	F	41	NF

M: male, F: female.

**Table 2:** Ten best-performing neural network models for diagnostic performance of miRNA combinations. Best-performing combination is indicated in bold.

Model Combination	TP	FP	TN	FN	Specificity	Sensitivity
***miR_106b*** ** + *miR_130b_3p* + *miR_127_3p* + *miR_129_5p* + *miR_30d_5p***	14.92	4.34	14.92	2.07	77.44	87.82
*miR_106b* + *miR_130b_3p* + *miR_127_3p* + *miR_129_5p* + *miR_671_5p* + *miR_30d_5p*	14.86	6.03	14.86	2.13	71.11	87.47
*miR_106b* + *miR_130b_3p* + *miR_127_3p* + *miR_769_5p* + *miR_671_5p* + *miR_30d_5p*	14.54	5.56	14.54	2.45	72.33	85.56
*miR_769_5p* + *miR_375_3p* + *miR_30d_5p*	13.85	4.44	13.85	3.14	75.70	81.52
*miR_106b* + *miR_130b_3p* + *miR_127_3p* + *miR_769_5p* + *miR_30d_5p*	14.59	5.97	14.59	2.40	70.96	85.88
*miR_106b* + *mir_194_5p* + *miR_130b_3p* + *miR_127_3p* + *miR_129_5p* + *miR_30d_5p*	14.16	5.39	14.16	2.83	72.42	83.33
*miR_106b* + *miR_130b_3p* + *miR_127_3p* + *miR_769_5p* + *miR_671_5p*	14.00	5.29	14.00	2.99	72.567	82.4
*miR_106b* + *miR_130b_3p* + *miR_127_3p* + *miR_129_5p* + *miR_769_5p* + *miR_30d_5p*	14.65	6.84	14.65	2.34	68.14	86.19
*miR_106b* + *miR_130b_3p* + *miR_127_3p* + *miR_671_5p* + *miR_30d_5p*	14.20	6.12	14.20	2.79	69.85	83.53
*miR_106b* + *miR_130b_3p* + *miR_127_3p* + *miR_129_5p* + *miR_375_3p*	14.93	7.85	14.93	2.06	65.52	87.84

FN: false negative; FP: false positive; TN: true negative; TP: true positive.

## Data Availability

NGS data were uploaded to an openly accessible database under Gene Expression Omnibus accession number GSE265752.

## Supplementary Materials

The following supporting information can be downloaded at: https://www.mdpi.com/article/10.3390/cancers16142528/s1, Table S1: NGS miRNA combined data; Table S2: Diagnostic performance of miRNA combinations derived from neural network models.
